# Long Working Hours and Subsequent Use of Psychotropic Medicine: A Study Protocol

**DOI:** 10.2196/resprot.3301

**Published:** 2014-09-19

**Authors:** Harald Hannerz, Karen Albertsen

**Affiliations:** ^1^National Research Centre for the Working EnvironmentCopenhagenDenmark; ^2^TeamArbejdsliv ApSCopenhagenDenmark

**Keywords:** occupational health, working time arrangements, mental health, prescription drugs, psychotropic medicine

## Abstract

**Background:**

Mental ill health is the most frequent cause of long-term sickness absence and disability retirement in Denmark. Some instances of mental ill health might be due to long working hours. A recent large cross-sectional study of a general working population in Norway found that not only “very much overtime”, but also “moderate overtime” (41-48 work hours/week) was significantly associated with increased levels of both anxiety and depression. These findings have not been sufficiently confirmed in longitudinal studies.

**Objective:**

The objective of the study is to give a detailed plan for a research project aimed at investigating the possibility of a prospective association between weekly working hours and use of psychotropic medicine in the general working population of Denmark.

**Methods:**

People from the general working population of Denmark have been surveyed, at various occasions in the time period 1995-2010, and interviewed about their work environment. The present study will link interview data from these surveys to national registers covering all inhabitants of Denmark. The participants will be followed for the first occurrence of redeemed prescriptions for psychotropic medicine. Poisson regression will be used to analyze incidence rates as a function of weekly working hours (32-40; 41-48; > 48 hours/week). The analyses will be controlled for gender, age, sample, shift work, and socioeconomic status. According to our feasibility studies, the statistical power is sufficient and the exposure is stable enough to make the study worth the while.

**Results:**

The publication of the present study protocol ends the design phase of the project. In the next phase, the questionnaire data will be forwarded to Statistics Denmark where they will be linked to data on deaths, migrations, socioeconomic status, and redeemed prescriptions for psychotropic medication. We expect the analysis to be completed by the end of 2014 and the results to be published mid 2015.

**Conclusions:**

The proposed project will be free from hindsight bias, since all hypotheses and statistical models are completely defined, peer-reviewed, and published before we link the exposure data to the outcome data. The results of the project will indicate to what extent and in what direction the national burden of mental ill health in Denmark has been influenced by long working hours.

## Introduction

### Mental Health and Long Working Hours

Mental health problems have become the biggest single cause for a disability benefit claim in the Organization for Economic Cooperation and Development countries. In Denmark, they account for almost 50% of all new claims [1]. The research literature suggests that some instances of mental ill health might be due to long working hours. It has been shown that long working hours are associated with short sleep [2-4] and fatigue [2,4-6], and that both of these conditions are predictors of psychiatric disorders [7-13]. There are, however, also some prophylactic effects of long working hours. All else being equal, long working hours would increase income, and thereby, decrease the risk of financial strain, which is a condition proven to be highly predictable of psychiatric disorders [14-16]. Some individuals who work long hours appear to be in a situation where there is an imbalance between the prophylactic and the pathogenic effects of the prolonged work [17]. From this viewpoint, some cases of mental ill health would be caused by long working hours, while others would be prevented in that prolonged work might solve problems which otherwise would lead to mental ill health.

### The Danish Government and Increased Working Hours

The Danish government wants to increase the total number of work hours in the nation by: (1) decreasing the number of hours that are lost due to sickness absence and early retirement, and (2) encouraging the ones that are working, to work longer hours [18]. From this perspective, we want to know if there is a positive, negative, or no correlation between long working hours and incidence of psychiatric disorders in the Danish working population, in other words, if pathogenic effects of long working hours outweighs the salutogenic effects or vice versa. We are especially interested in the situation among the people who perform moderate overtime work (41-48 work hours/week), that is, overtime work which lies within the limits of the European working time directive. If there is a nonnegligible correlation between long working hours and subsequent mental ill health, then this needs to be taken into account in the planning and evaluation of strategies aimed at getting more people to work extra hours.

The existing literature does not provide the information we need to settle this question.

### Studies and Findings

A recent large cross-sectional study of a general working population in Norway found that not only “very much overtime” (49-100 work hours/week), but also “moderate overtime” (41-48 work hours/week) was significantly associated with increased levels of both anxiety and depression [19]. These findings have, however, not been sufficiently confirmed in studies that utilize longitudinal designs. The hypothesis that “very much overtime” is a predictor of mental disorders is supported by three prospective company based studies [20-22]. Suwazono et al [20] focused on the development of mental disorders among workers in a large telecommunication enterprise in Japan. They found that the odds for having developed mental disorders at the end of a four year follow-up period were higher among those who worked > 12 hours/day at baseline than they were among those who worked ≤ 8 hours/day. The estimated odds ratio was statistically significant among the men, but not among the women. Virtanen et al [22] focused on the development of depression and anxiety symptoms in a group of middle-aged British civil servants. They found that those who worked more than 55 hours/week at baseline, compared with those who worked 35-40 hours/week, had a higher propensity for developing depression and anxiety symptoms during an approximately five year follow-up period. Based on the same database, it has also been shown that the civil servants who worked more than 55 hours/week had a higher propensity for developing sleeping disorders [21]. The latter result is, however, contradicted by a large population-based prospective study wherein the odds for having developed sleeping disorders at the end of a five year follow-up period were lower among those who often worked more than 48 hours/week at baseline than they were among other employees [23].

The company-based studies mentioned above also compared the effect of “moderate overtime” (defined as 8-12 work hours/day by Suwazono et al, and 41-55 work hours/week by Virtanen et al) [20-22] with the effect of “normal working hours” (defined as ≤ 8 work hours/day by Suwazono et al, and 35-40 work hours/week by Virtanen et al) [20-22]. The studies suggest a tendency for higher risks among those with moderate overtime work, but the contrast is not statistically significant in any of the studies.

### Aim and Hypothesis

The null-hypothesis of the present project is that long working hours, to the extent that it is currently practiced in Denmark, neither adds to nor subtracts from the national burden of mental ill health.

The project aims at testing the above hypothesis in a prospective cohort study on a random sample of the Danish working population. The null-hypothesis will be rejected if subsequent rates of mental ill health (manifested by the use of psychotropic medicine) among people with long working hours at baseline differ significantly from those among people with normal working hours. The analysis will be adjusted for gender, age, socioeconomic status, and shift work. It will only include those who are free from symptoms at baseline.

## Methods

### Ethics Approval

The study will comply with The Act on Processing of Personal Data (Act No. 429 of May 31, 2000), which implements the European Union Directive 95/46/EC on the protection of individuals.

### Data Material

The data material of the project will be obtained through a linkage of data from the Copenhagen Psychosocial Questionnaire (COPSOQ) study sample of 2004, the Danish National Working Environment Survey (DANES) of 2008, and the Danish Work Environment Cohort Study (DWECS) of 1995, 2000, 2005, and 2010 with data from the Central Person Register (CPR), the Employment Classification Module (ECM), and the Danish National Prescription Registry (DNPR). The participants’ unique personal identification numbers will be used as the key in the linkage procedure. DNPR covers all purchases of prescription drugs at pharmacies in Denmark since 1995 [24]. The CPR has existed since 1968, and contains dates of deaths and migrations in the Danish population [25]. A person’s occupation, industry, and socioeconomic status are, as of 1975, registered annually in the ECM [26]. Socioeconomic status is coded in accordance with Statistics Denmark’s official socioeconomic classification [27]. The socioeconomic status (SES) code among employees is based on the first digit of the Danish version of the International Standard Classification of Occupations (DISCO-88) [28], and contains the following categories: (1) legislators, senior officials, and managers (DISCO-88 group 1), (2) professionals (DISCO-88 group 2), (3) technicians and associate professionals (DISCO-88group 3), (4) workers in occupations that require skills at a basic level (DISCO-88 group 4-8), (5) workers in elementary occupations (DISCO-88 group 9), and (6) gainfully occupied people with an unknown occupation (missing DISCO-88 code).

The COPSOQ study sample is a random sample, which comprises 4732 people, 20-59 years of age, whereof 3517 are wage earners [29]. DANES is based on a random sample of the Danish population in 2008. It contains responses from 6531 people 18-59 years of age, of which 4919 are employees. The DWECS is an open cohort study, which was initiated in 1990 with a random sample of people 18-59 year of age in the Danish population. The cohort has thereafter been supplemented with young people and immigrants so as to obtain a representative cross-sectional study of at least 5000 employees every fifth year [30].

The reported response rates were 80, 75, 60, 62, 66, and 48 percent for DWECS 1995 [31]; DWECS 2000 [31]; COPSOQ 2004 [29]; DWECS 2005 [32]; DANES 2008 [33]; and DWECS 2010 [34], respectively. These response rates are, however, only correct for the data that were collected prior to 2000. In May 2000, a new law was passed which made it easier for citizens to be registered as permanent nonrespondents in research surveys based on random samples from the central person register; a registration which protects them from being contacted by researchers. The reported response rates above do not take the registered nonrespondents into account. For example, 14% of all employees in Denmark were registered as permanent nonrespondents in 2008 [35]. If we take people from this group into account then the response rate for the DANES survey in 2008 will fall from the reported 65.88% (6531/9913) to 56.66% (6531/11,527).

The COPSOQ, the DANES, and the DWECS surveys contain person-based information on weekly working hours, calculated by adding the hours worked in secondary jobs to the ones in a primary job. The wording of the questions differs, however, slightly between the various questionnaires. The DWECS questionnaires of 1995, 2000, and 2005 ask for weekly working hours in current jobs or (if the person is momentarily out of work) in the last held job. DWECS 2010 asks for current weekly working hours without further specification. COPSOQ and DANES ask for average working hours during the one year period preceding the time of the interview. The COPSOQ questionnaire only allowed participants to report between 0 and 99 working hours per week, while the other questionnaires allowed an unlimited number of hours. Another peculiarity of the COPSOQ questionnaire is that it uses a single question to ask for the combined number of hours worked in primary and secondary jobs, while the other questionnaires use one question for the number of hours worked in the primary job and another one for the hours worked in secondary jobs. The surveys also contain information on the participants’ normal work schedules. Again, the questions and response categories vary slightly between the questionnaires, but all of them can identify workers who are either on fixed night shifts or rotational shift work schedules. The exact wordings (in Danish) of the used questions are given in the appendix (see Multimedia Appendix 1). A translation into English is given; see Multimedia Appendix 2.

### Primary Analysis

#### Case Definition

The medical products in the DNPR are coded in accordance with the Anatomical Therapeutic Chemical (ATC) system. In the present project, a person will become a case if and when he or she redeems a prescription for drugs in the ATC-code category N05 (psycholeptica) or N06 (psychoanaleptica). The psycholeptic category contains antipsychotics, anxiolytics, hypnotics, and sedatives, while the psychanaleptic category contains antidepressants, psychostimulants, and antidementia drugs.

#### Follow-Up and Inclusion Criteria

Each of the included samples will be followed for a period of two to five years, beginning at the start of the calendar year succeeding the one in which they were sampled. People should be between 21 and 59 years old at the start of the follow-up period and, according to the questionnaire, employed with 32 or more weekly work hours around the time of the interview, to be eligible for inclusion. The sample from DWECS 2010 will be followed for two years. The sample from the DANES 2008 will be followed for four years. The remaining samples will be followed for five years. A participant will be censored if and when he or she dies or emigrates. Person years at risk will be calculated for each participant. People who redeemed a prescription for a medication with an ATC-code that belongs to the case definition, during the calendar year preceding baseline, will not be included. A participant who reaches the clinical endpoint of the study will not be allowed to reenter the follow-up. In other words, there will be maximum one case per person.

#### Statistical Model

We will use Poisson regression to analyze incidence rates of redeemed prescriptions for psychotropic medicine as a function of weekly working hours (32-40; 41-48; > 48 hours/week). The analysis will be controlled for gender, age (10 year classes), sample (DWECS 1995; DWECS 2000; COPSOQ 2004; DWECS 2005; DANES 2008; and DWECS 2010), shift work (fixed night shifts or rotational shift work schedules vs other), and SES (legislators, senior officials and managers; professionals; technicians and associate professionals; workers in occupations that require skills at a basic level; workers in elementary occupations; and gainfully occupied people with an unknown occupation). With SES we mean socioeconomic status, according to the employment classification module, during the calendar year of the baseline interview. The logarithm of person years at risk will be used as offset. A likelihood ratio test will be used to test the null-hypothesis, which states that the analyzed rates are independent of weekly working hours. The significance level is set to 0.05.

#### Power Calculations

Based on rates calculated through DNPR in the time period 2001-2005, we expect on average approximately 25 new cases per 1000 person years at risk (PYRS). We expect that the four follow-up periods of nonprevalent cases in the DWECS samples will provide approximately 68,000 PYRS, the five year follow-up of the COPSOQ sample will provide 12,000 PYRS, and the four year follow-up of the DANES sample will provide 16,000 PYRS. Among the ones who worked 32 or more hours per week in 2000 according to DWECS, 26.7% worked more than 40 hours/week and 10.5% worked more than 48 hours/week.

**Figure 1:**
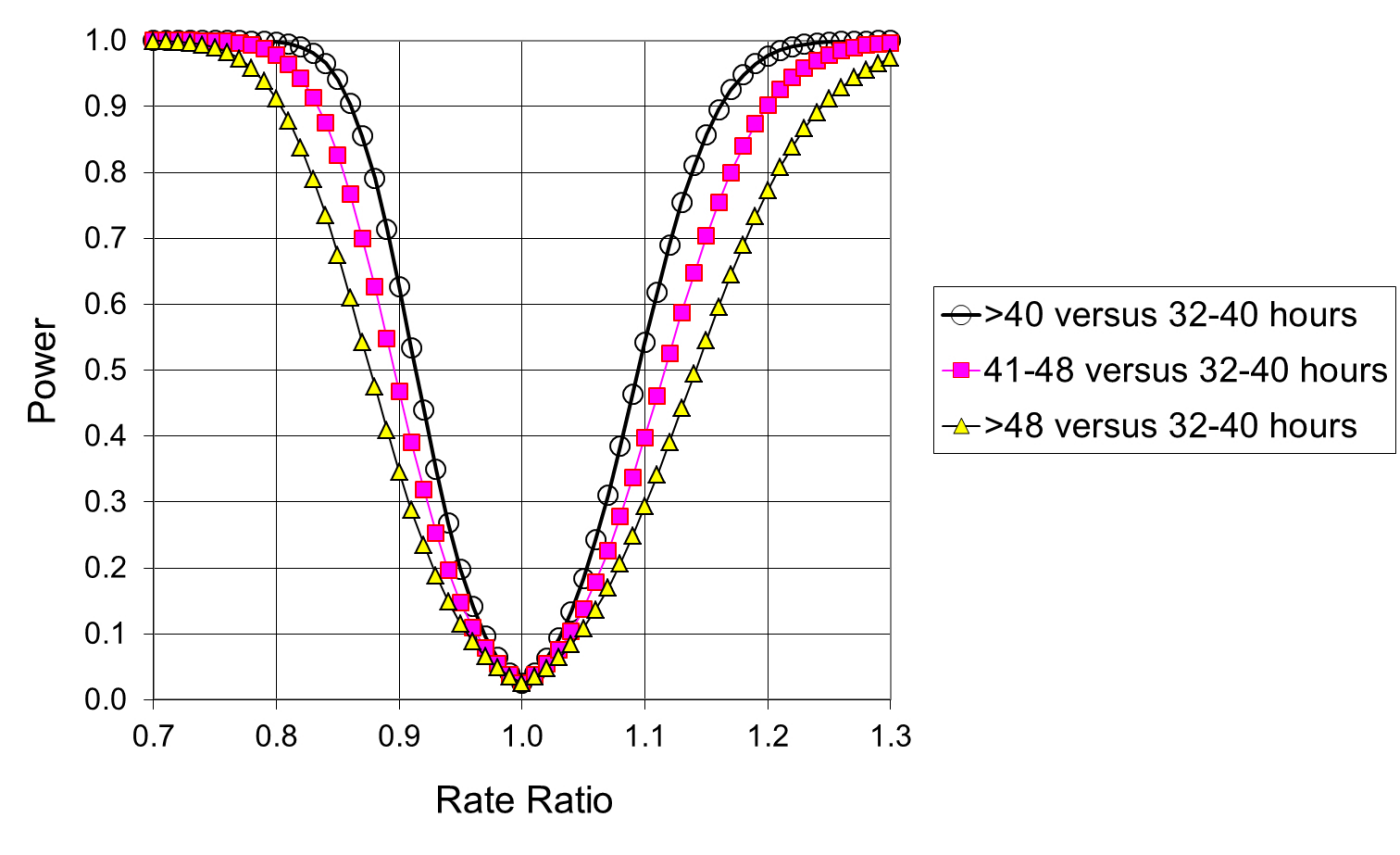
Power of detecting differences in psychiatric morbidity rates in Denmark, between overtime workers and people with normal work hours, as a function of the true rate ratio.

With the above data as input, Figure 1 illustrates the statistical powers to detect contrasts between normal working hours (32-40 hours/week) on one hand and “overtime work” (>40 work hours/week), “moderate overtime work” (41-48 work hours/week), and “very much overtime” (49-100 work hours/week) on the other, as a function of the true rate ratio. If, for example, the true rate ratio between moderate overtime workers and those with normal working hours is 1.2 then we have a 90% chance of detecting the difference; if it is 1.3 then the chance of detection is 99.7%. The power calculations are based on the Poisson distribution, the propagation of error formulas, and the central limit theorem.

#### Transitions Between Work Categories

The subjects of our study will be categorized according to their exposure status at baseline. They will thereafter be followed for five years. We do not have information about their work schedules during the follow-up, but wanted to ascertain that the exposure statuses were stable enough to make the study worth the while. To check this, we used DWECS data to cross-tabulate a worker’s exposure status in 2000 with that in 2005. We included all who were economically active and 30 years or older in both waves. The exposure statuses were fairly stable over time; 68.3% (529/774) of the workers with long working hours and 83.28% (1240/1489) of the workers with normal working hours in 2005 had the same working conditions five years earlier, while 67.9% (529/778) of the workers with long working hours and 83.50% (1240/1485) of the workers with normal working hours in 2000 had the same working conditions five years later. Cohen’s kappa for agreement between the working hour category in 2000 and in 2005 was 0.52 (95% CI 0.48-0.55), which indicates a moderate agreement according to Landis and Koch [36].

### Secondary Analyses

Regardless of whether or not the primary research hypothesis is confirmed, we will perform a series of secondary analyses. The interpretation of the results from these analyses will, however, depend on the outcome of the primary hypothesis test. If the primary null-hypothesis is rejected, then the secondary analyses will be regarded as nested hypothesis tests. Otherwise, they will be regarded as hypothesis generating exploratory analyses, whose results need to be confirmed in an independent dataset before they can be deemed statistically significant.

With the endpoint and covariates of the primary hypothesis test, we will use likelihood ratio tests to check for possible two-way interaction effects between working hours and gender, age, shift work (fixed night shifts or rotational shift work schedules vs other), or socioeconomic status. Subsample analyses will thereafter be performed on data stratified, first by gender, then by age (21-39; 40-59 years old), then by shift work, and finally by socioeconomic status. In keeping with the principles of nested hypothesis testing, perceived differences in effect sizes between strata will not be considered statistically significant unless both the primary hypothesis test and the concerned two-way interaction effect are statistically significant.

In addition to the above, we will perform three separate analyses, in exactly the same way as we did in the primary analysis, but with endpoints defined by the following subsets of ATC-codes, N05B (anxiolytics), N05C (hypnotics and sedatives), and N06A (antidepressants).

### Auxiliary Analysis Regarding Prescription Bias

Some people with sleeping disorders seek medical care to cope with the situation, while others do not. The same holds for anxiety and depressive disorders. We can therefore not know if an increased use of medicine is due to an increased need of treatment or an increased propensity to seek treatment. Our primary analysis will show if long working hours is associated with subsequent use of psychotropic medicine. Such a finding is of interest in itself. Obtained rate ratios can, however, only be interpreted as morbidity rate ratios if we can show that prescription bias is likely to be negligible.

Our data material allows us to evaluate whether or not prescription bias is present. The questionnaires that we describe in our project plan contain questions on mental health, which enable us to relate the prevalence of self-rated mental ill health to the prevalence of medicine users among people with long and normal working hours respectively.

In particular, the DANES questionnaire and the DWECS questionnaires of 1995, 2000, and 2005 contain all items needed to score the participants on the five item scale of the mental health inventory MHI-5, which is a subscale of the SF-36 general health index [37]. The MHI-5 scale is based on the following questions, all relating to the past four weeks: (1) “Have you been very nervous?”; (2) “Have you felt so down in the dumps that nothing could cheer you up?”; (3) “Have you felt calm and peaceful?”; (4) “Have you felt down-hearted and depressed?”; and (5) “Have you been happy?”. Each question should be answered with one of the following response categories; “all of the time”; “most of the time”; “a good bit of the time”; “some of the time”; “a little of the time”; or “none of the time”. The categories are scored, in the order listed, from 1 to 6 for the first, second, and fourth question, and from 6 to 1 for the third end fifth question. The score of the full scale is a function of the scores of the items, and ranges between 0 and 100. The equation reads y=100(x - n)/(5n), where y is the score of the full scale, n is the number of included items, and x is the sum of the scores of the included items. If the response is missing for two items or more then the whole scale will be categorized as missing. A lower score on the full scale indicates a poorer mental health, and a score which is less than or equal to 52 is regarded as an indicator of severe mental health problems [33,38,39]. The Danish translation of the SF-36 questionnaire has been validated [40] and the MHI-5 subscale has been shown to be reliable in the general population of Denmark (Cronbach alpha = 0.80) [41].

To shed some light on the matter of prescription bias, we will perform an auxiliary cross-sectional analysis, which compares odds ratios for poor self-rated mental health with odds ratios for use of psychotropic medicine among workers with long versus normal working hours. If either of these ratios is statistically significant, while the other one points in the opposite direction, then the idea that an increased/decreased rate of medicine usage can be interpreted as an increased/decreased rate of poor mental health is contradicted.

For this analysis, we will not include information from COPSOQ or DWECS 2010. Otherwise, the data sources are the same as the ones described for the primary analysis. Prevalent cases will not be excluded, but the rest of the inclusion/exclusion criteria will be the same as the ones of the primary analysis. The following case definitions will be employed for poor self-rated mental health and use of psychotropic medicine, respectively: (1) a score on the mental health inventory (MHI-5) that is less than or equal to 52, and (2) redemption of a prescription for a drug in the ATC-code category N05 (psycholeptica) or N06 (psychoanaleptica), during the calendar year of the interview.

Logistic regression will be used to model the odds of the outcomes as a function of weekly working hours (>40 hrs/week; 32-40 hrs/week). The analysis will be controlled for gender, age, socioeconomic status, shift work, and sample in the same way as was done in the primary analysis. Generalized estimating equations will be used to estimate the parameters. Observations from the same person will be treated as repeated measurements. A first order autoregressive correlation structure is assumed. The odds ratio (OR) between the exposed and the nonexposed will be calculated and presented with a 95% CI. The CI will be based on the empiric standard error. An OR will be considered statistically significant if the CI does not contain 1.

Approximately 16,500 employees will fulfill the inclusion criteria of this auxiliary analysis. The prevalence of poor self-rated mental health (MHI-5 ≤ 52) among Danish employees was estimated to 7.3% in 2008 [33], while the corresponding prevalence for use of psychotropic medicine was 9.3% (according to a linkage between the ECM and the DNPR). With such a frequency of events, we estimate that the power to detect an OR of 1.2 or higher or 1/1.2 or lower between employees with long versus normal working hours will be at least 80% for either of the two outcomes.

### Sensitivity Analyses

#### Exclusion of Workers With Poor Self-Rated Mental Health at Baseline

Workers who redeemed a prescription for psychiatric drugs in the year preceding baseline are excluded from the study. This is done to counteract the possibility of a healthy-worker bias. There is, however, a possibility of residual confounding, since mental health problems may exist also among workers who do not use prescription drugs. We want to know to what extent and in what direction the estimates to be obtained in the primary analysis would change if we were able to exclude all workers with mental health problems at baseline, regardless of whether or not they redeemed prescription drugs in the year preceding baseline. We will address this issue with a sensitivity analysis, which, in addition to excluding the participants who were prescribed medication, also excludes those with poor self-rated mental health at baseline, according to MHI-5 (score ≤ 52). Due to a discrepancy in the response categories of the MHI-5 questions, the sensitivity analysis cannot include data from COPSOQ 2004 and DWECS 2010. In all other respects, we will use the same statistical model as we did in the primary analysis. The parameters will be estimated first with medicine usage only, and then with medicine usage and/or poor self-rated mental health at baseline as exclusion criteria.

#### Are the Estimates Influenced By Job Satisfaction and Job Insecurity?

It has been shown that the psychosocial work environment is associated with mental health. According to a meta-analytic review by Stansfeld and Candy, common mental disorders were predicted by job strain, low decision latitude, low social support, high psychological demands, effort-reward imbalance, and high job insecurity [42]. Another finding, of particular interest to the present study, is a statistically significant relation between baseline job dissatisfaction and subsequent usage of psychiatric prescription drugs in a recent sample of 40-60 year old employees of the City of Helsinki [43].

Job satisfaction does not measure the work environment directly. It has, however, been shown to be highly dependent on the psychosocial work environment. The COPSOQ scales on demands, influence and development, interpersonal relations, and leadership explained 59% of the variation in job satisfaction, in a sample of employees in Germany 2004 [44]. Further, a meta-analysis based on 485 studies suggested that job satisfaction, with an overall correlation of 0.37 across four health outcomes, was an important factor influencing the health of workers [45].

Unfortunately, the questions and response categories that deal with psychosocial dimensions differ between our questionnaires to a degree that make it impossible to obtain any psychosocial measure that stays the same in all of the datasets. The questions and response categories for job satisfaction and job insecurity remained, however, unchanged throughout the questionnaires of DWECS 1995, 2000, and 2005, and this subset of the data is large enough to make a sensitivity analysis meaningful.

We want to know how and to what extent we can expect our primary analysis to be influenced by uncontrolled differences in the psychosocial work environment. We will address this issue with a sensitivity analysis, where we perform the working hour analysis on the above-mentioned subset of the data, both with and without control for job insecurity and job satisfaction. The following questions will be used: (1) “Are you worried about becoming unemployed?”; (2) “Are you worried about difficulties in finding a new job with your present qualification?”; and (3) “Are you satisfied with your job?”. The questions on job insecurity (which could be answered with either “Yes” or “No”) will be combined into one dichotomous variable. Participants who answered “Yes” to at least one of the two questions will be categorized as having job insecurity, while those who answered “No” to both questions will be categorized as not having job insecurity. Job satisfaction will be treated as a categorical variable in three levels: (1) “To a high degree”; (2) “To some degree”; and (3) “Only to a lesser degree” or “No, or only to a slight degree”. The working time arrangement parameters will be estimated first with, and then without, control for job insecurity and job satisfaction. In all other respects, we will use the statistical model of the primary analysis.

### Shift Work

#### The Effect of Shift Work on Health

We control for shift work in the primary, the auxiliary, and all of the secondary analyses. In doing so, we will automatically obtain parameter estimates that can be used to calculate rate ratios for psychotropic medicine as a function of shift work (fixed night shifts or rotational shift work schedules vs other). To our knowledge, the prospective relationship between shift work and psychotropic medicine has never been studied in a general working population. Hence, the effect of shift work will be of interest, not only as a control variable, but also as a potential contribution to the body of knowledge on “shift work and health”. In our opinion, the data material, inclusion criteria, significance levels, and statistical models that we use to estimate effects of long working hours are appropriate also for the estimation of shift work effects. Moreover, the primary statistical power is sufficient and the exposure is stable enough to make such an effort worth the while (see below). We will therefore publish results on shift work in addition to the ones on long working hours.

#### Transitions Between Work-Schedule Categories

We used DWECS data to cross-tabulate an employee’s work schedule category in 2000 with that in 2005. We included all who were economically active and 30 years or older in both waves. More than half (55.1%) (113/205) of the workers with shift work and 90.90% (1858/2044) of the workers with nonshifting work hours in 2005 had the same type of work schedule five years earlier, while 37.8% (113/299) of the workers with shift work and 95.28% (1858/1950) of the workers with nonshifting work hours in 2000 had the same type of work schedule five years later. Cohen’s kappa for agreement between the work schedule category in 2000 and in 2005 was 0.38 (95% CI 0.32-0.44), which indicates fair agreement according to Landis and Koch [36].

#### Power Calculations for the Shift Work Hypothesis

**Figure 2:**
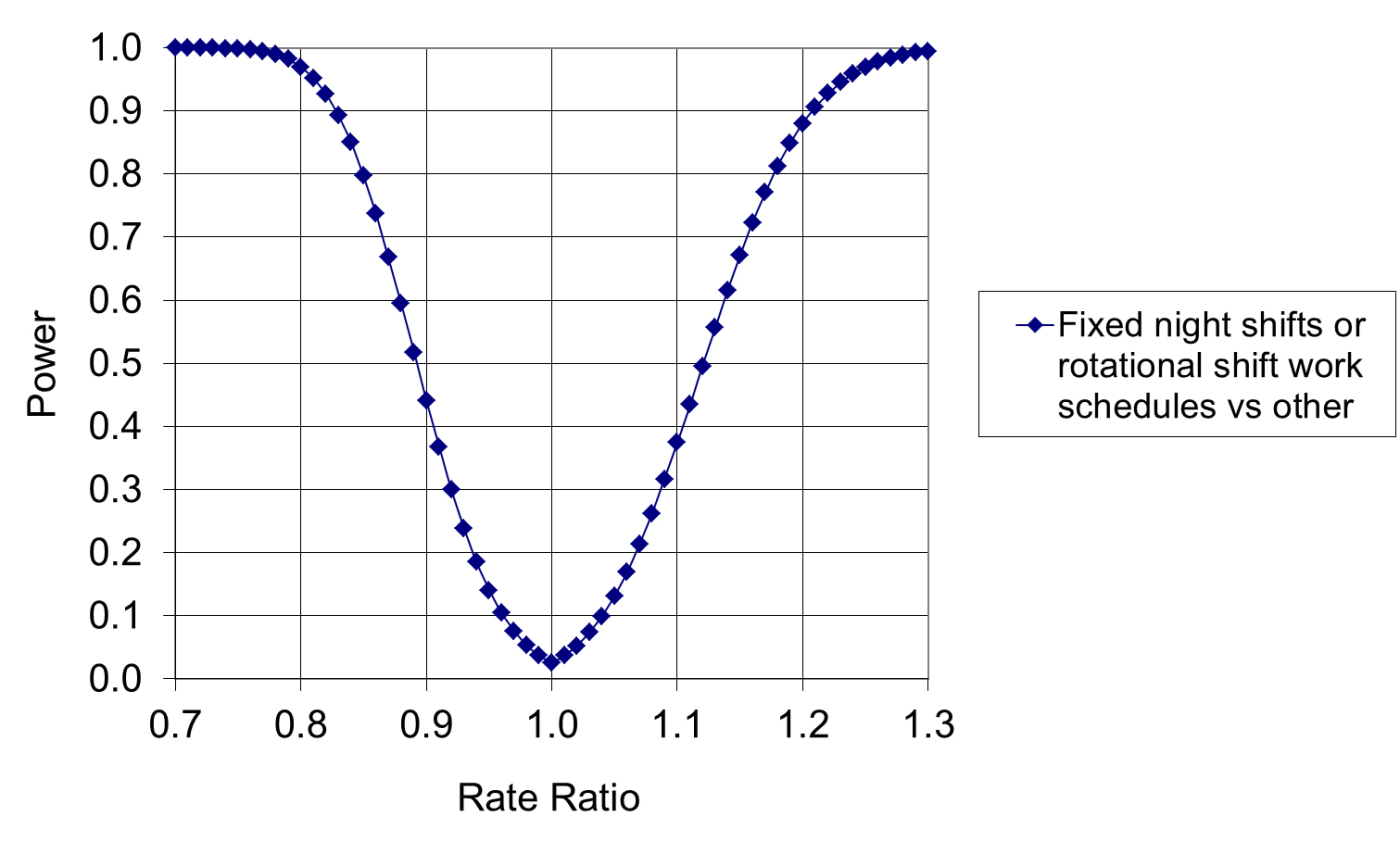
Power of detecting differences in psychiatric morbidity rates in Denmark, between employees on fixed night shifts or rotational shift work schedules and employees with other work schedules, as a function of the true rate ratio.

According to DWECS 2000, 14.4% of the ones who worked 32 or more hours per week were either on fixed night shifts or rotational shift work schedules. We applied this information to the data and assumptions we used in the power calculation for the primary analysis and calculated the statistical power to detect a contrast between fixed night shifts or rotational shift work schedules on one hand, and nonshifting nonnight work on the other (Figure 2 shows this contrast).

## Results

The publication of the present study protocol ends the design phase of the project. In the next phase, the questionnaire data will be forwarded to Statistics Denmark where they will be linked to data on deaths, migrations, socioeconomic status, and redeemed prescriptions for psychotropic medication. We expect the analysis to be completed by the end of 2014 and the results to be published mid 2015.

## Discussion

### The Research Project

This protocol gives a detailed plan for a research project aimed at investigating the possibility of a prospective association between weekly working hours and use of psychotropic medication in the general working population of Denmark. We have shown that the statistical power is sufficient, and that the exposure is stable enough to make the study worth the while. We have also designed an auxiliary study, which will help us to interpret the results of the primary analysis, which, a priori, might be affected both by differences in morbidity and differences in propensity to seek treatment.

The presented study will utilize the work hour categories proposed by Kleppa et al [19], that is, 32-40 to represent normal weekly working hours, 41-48 to represent overtime work which lies within the limits of the European working time directive, and 49-100 to represent overtime work beyond the threshold of the directive. These cut-off points will enable us to evaluate the results from a societal (national burden of disease) perspective, since they allow us to answer the following questions, “Are there important differences between the average rate among people with normal working hours, and that among people with overtime work within and beyond the EU directive, respectively?”. The span of the maximum work hour category complicates, however, the interpretation of the results from an individual perspective. A null finding, which would indicate that the effect is unimportant from a societal perspective, does not necessarily mean that it is safe to work, for example, 60 hours or more per week. From this viewpoint, we understand that an extra work hour category would be of interest, especially to people in nations where it is normal to work 50 hours (or more) per week. An extra category would, however, reduce the statistical power of the analysis, and we prefer to have one sufficiently powered category (> 48 hours), instead of two underpowered categories (eg, 49-59; ≥ 60 hours).

Since our goal is to obtain statistical certainty, we choose to base our primary case definition on the aggregate of all types of psychotropic medicine, rather than performing analyses on particular types. It makes sense to do so. Anxiety disorders are strongly and positively correlated with depressive disorders [19,22], and sleeping disorders are strongly and positively correlated with both anxiety and depressive disorders [46]. Based on a linkage between the ECM and the National Prescription Registry, 95.83% (75,815/79,117) of all new cases of psychotropic medicine use among employees in Denmark in 2001 concerned a drug in the category anxiolytics (33.26%, 26,315/79,117, of all cases), antidepressants (30.50%, 24,131/79,117, of all cases), or hypnotics and sedatives (32.07%, 25,369/79,117, of all cases).

It also makes sense to control for gender, age, socioeconomic status, and shift work. It is well established that women are treated for depression [47], anxiety [48,49], and insomnia [50] more often than men and that the occurrence of these diagnoses varies with age [47,49,50]. It is also well established that socioeconomic status is negatively correlated with mental illness; the lower the SES, the higher the rate of illness [51]. Shift work may induce the so-called shift work sleep disorder, and it is possible that such a condition will be treated with hypnotics as well as wakefulness-promoting drugs [52].

### Study Strengths and Weaknesses

Since the clinical endpoint of the study is determined through national registers, which cover all residents of Denmark, and we are able to censor for deaths and emigrations, we have eliminated bias from missing follow-up data. The study is further strengthened by its prospective design, the exclusion of prevalent cases and the use of a study population that has been randomly sampled from the target population. Another advantage is that all hypotheses and statistical models are completely specified, peer reviewed, and published before we merge the questionnaire data to the registers.

Nonrespondents in the baseline interviews weaken the study. Since long working hours imply less time to answer questionnaires, the response rates as well as the reasons for nonresponse might differ between the exposed and the unexposed workers. Selection bias cannot be ruled out. We believe, however, that any such bias will be mitigated through the exclusion of prevalent cases. Another weakness is the lack of adequate information on sleeping habits at baseline. As mentioned in the Introduction, one of the main theoretic reasons for an adverse effect of long working hours is its association with short sleep, which in turn is a predictor of mental disorders. If there were an effect of long working hours on mental health, then it would have been interesting to know to what extent this effect could be attributed to sleeping habits.

The study is not a randomized controlled trial, and can therefore not confirm etiological hypotheses. It may, however, confirm that long working hours is a predictor for incident use of psychotropic medicine, and if so, it will lend support to the hypothesis of a causal relationship.

## Multimedia Appendix 1

The wording (in Danish) of the questions used to obtain information on working hours and work schedules.

## Multimedia Appendix 2

The wording (translated from Danish) of the questions used to obtain information on working hours and work schedules.

## Abbreviations

ATC: Anatomical Therapeutic Chemical
COPSOQ: Copenhagen Psychosocial Questionnaire
CPR: Central Person Register
DANES: Danish National Working Environment Survey of 2008
DISCO: Danish version of the International Standard Classification of Occupations
DNPR: Danish National Prescription Registry
DWECS: Danish Work Environment Cohort Study of 1995, 2000, 2005, and 2010
ECM: Employment Classification Module
MHI-5: mental health inventory
NRCWE: National Research Centre for the Working Environment
OR: odds ratio
PYRS: person years at risk
SES: socioeconomic status
