# Supplementary material for: Long Working Hours and Subsequent Use of Psychotropic Medicine: A Study Protocol
Source: JMIR Res Protoc. 2014 Sep 19;3(3):e51. doi: 10.2196/resprot.3301 (PMC4180344; doi:10.2196/resprot.3301)
Supplement: Supplementary file 1 [file resprot_v3i3e51_app1.pdf]

APPENDIX 1: THE WORDING (IN DANISH) OF THE QUESTIONS USED TO OBTAIN INFORMATION ON WORKING HOURS AND WORK SCHEDULES

Weekly working hours:

DWECS, 1995

(De næste spørgsmål handler om Deres nuværende arbejde. Hvis De har flere jobs, vil vi gerne spørge om Deres hovedbeskæftigelse som lønmodtager (den de bruger flest timer på). Hvis De ikke er i arbejde i øjeblikket, beder vi Dem svare vedrørende Deres seneste job.)

Hvor mange timer om ugen arbejder De normalt i Deres hovedjob som lønmodtager? Antal timer om ugen:______

Og i Deres bijob? Antal timer om ugen:______

(Hovedjobbet er det job. IP bruger flest timer på uanset om det er 10 eller 37 timer. Hvis flere bijob, tælles alle timerne i bijob sammen. Hvis timeantallet varierer, må det spørges om et omtrent gennemsnit over en måned.)

DWECS, 2000

(De næste spørgsmål handler om dit nuværende arbejde som lønmodtager. Hvis du har flere jobs, vil vi gerne spørge om din hovedbeskæftigelse som lønmodtager (den du bruger flest timer på). Hvis du ikke er i arbejde i øjeblikket, beskriv dit seneste job.)

Hvor mange timer om ugen arbejder du normalt i dit hovedjob, inkl. eventuelle fastlagte timer, betalt overarbejde og andet ekstraarbejde, fx hjemmearbejde? Angiv timer i gennemsnit: _ _ _

Hvor mange timer om ugen arbejder du normalt i dit bijob, inkl. eventuelle fastlagte timer, betalt overarbejde og andet ekstraarbejde, fx hjemmearbejde? Angiv timer i gennemsnit: _ _ _

DWECS, 2005

(De næste spørgsmål handler om dit nuværende arbejde som lønmodtager. Hvis du har flere jobs, vil vi gerne at du beskriver din hovedbeskæftigelse som lønmodtager (den du bruger flest timer på). Hvis du ikke er i arbejde i øjeblikket, beskriv dit seneste job.)

Hvor mange timer om ugen arbejder du normalt i din hovedbeskæftigelse, inkl. eventuelle fastlagte timer, betalt overarbejde og andet ekstraarbejde, fx hjemmearbejde? I gennemsnit _ _ _ timer _ _ minutter

Hvor mange timer om ugen arbejder du normalt i din bibeskæftigelse, inkl. eventuelle fastlagte timer, betalt overarbejde og andet ekstraarbejde, fx hjemmearbejde? I gennemsnit _ _ _ timer

DWECS, 2010
Hvor mange timer om ugen arbejder du i din hovedbeskæftigelse, inkl. eventuelle ekstratimer? Antal hele timer _ _ _

Hvor mange timer om ugen arbejder du normalt i din bibeskæftigelse? Antal hele timer _ _ _

COPSOQ, 2004
Hvor mange timer arbejder du rent faktisk om ugen, når du tæller overarbejde og bijob med? (Gennemsnit per uge i det seneste år) _ _ timer per uge

DANES, 2008
Hvor mange timer arbejder du rent faktisk om ugen i din hovedbeskæftigelse? (Skriv venligst gennemsnit per uge i det sidste år) _ _ _ timer per uge

Hvis du har et bijob, hvor mange timer arbejder du så rent faktisk om ugen i dit bijob? (Skriv venligst gennemsnit per uge i det sidste år) _ _ _ timer per uge

Work schedules:

DWECS, 1995
Hvordan er Deres arbejdstid normalt placeret? (Hovedbeskæftigelsen som lønmodtager)
Fast dagtid; Toholdsskift; Treholdsskift; Uregelmæssig placering i løbet af døgnet/ugen efter særligt arbejdsskema eller turnusordning; Fast aftenskift/aftenarbejde; Fast natskift/natarbejde; Fast morgenarbejde; Andet.

DWECS, 2000
Hvordan er din arbejdstid normalt placeret? (gælder hovedbeskæftigelsen)
Fast daghold; Toholdsskift; Treholdsskift; Uregelmæssig placering i løbet af døgnet/ugen efter særligt arbejdsskema eller turnusordning; Fast aftenskift/aftenarbejde; Fast natskift/natarbejde; Fast morgenarbejde; Andet.

DWECS, 2005

Hvordan er din arbejdstid normalt placeret? (gælder hovedbeskæftigelsen)
Dagtid eller fast daghold; Toholdsskift; Treholdsskift; Uregelmæssig placering i løbet af døgnet/ugen efter særligt arbejdsskema eller turnusordning; Fast aftenskift/aftenarbejde; Fast natskift/natarbejde; Fast morgenarbejde; Andet.

DWECS, 2010
På hvilket tidspunkt af døgnet arbejder du sædvanligvis i din hovedbeskæftigelse?
Fast dagarbejde; Fast aftenarbejde (overvejende mellem kl. 15 og 24); Fast natarbejde (overvejende mellem kl. 24 og 05); Skiftende arbejdstider med natarbejde; Skiftende arbejdstider uden natarbejde; Andet.

COPSOQ, 2004
På hvilket tidspunkt af døgnet arbejder du sædvanligvis?
Fast dagarbejde (overvejende mellem kl. 06 og 18); Fast aftenarbejde (overvejende mellem kl. 15 og 24); Fast natarbejde (overvejende mellem kl. 22 og 06); Skiftende arbejdstider uden natarbejde; Skiftende arbejdstider med natarbejde; Andet.

DANES, 2008
På hvilket tidspunkt af døgnet arbejder du sædvanligvis i din hovedbeskæftigelse?
Fast dagarbejde (overvejende mellem kl. 06 og 18); Fast aftenarbejde (overvejende mellem kl. 15 og 24); Fast natarbejde (overvejende mellem kl. 22 og 06); Skiftende arbejdstider; Andet.
