# Supplementary material for: Long Working Hours and Subsequent Use of Psychotropic Medicine: A Study Protocol
Source: JMIR Res Protoc. 2014 Sep 19;3(3):e51. doi: 10.2196/resprot.3301 (PMC4180344; doi:10.2196/resprot.3301)
Supplement: Supplementary file 2 [file resprot_v3i3e51_app2.pdf]

APPENDIX 2: THE WORDING (TRANSLATED FROM DANISH) OF THE QUESTIONS USED TO OBTAIN INFORMATION ON WORKING HOURS AND WORK SCHEDULES

Weekly working hours:

DWECS, 1995

(The next questions are about your present work. If you have more than one job, then we only ask about your primary job as a wage earner (the job in which you work most hours). If you are not working at present, we kindly ask you to answer on basis of you last job.)

How many hours per week do you work in your primary job as a wage earner?
Number of hours per week: _________

How many hours per week do you work on the sideline?
Number of hours per week: __________

(The primary job is the job the IP spent most hours doing no matter if it is 10 or 37 hours. If he/she has several extra jobs, then the number of hours in these jobs must be added. If the number of hours varies, ask about an approximate monthly average.)

DWECS, 2000

(The next questions are about your present work. If you have more than one job, then we only ask about your primary job as a wage earner (the job in which you work most hours). If you are not working at present, we kindly ask you to answer on basis of you last job.)

How many hours per week do you work in your primary job as a wage earner?
(including scheduled hours, paid over-time, and other extra work, e.g. home work)
Average number of hours: ______

How many hours per week do you normally work on the sideline, including scheduled hours, paid overtime, and other extra work, e.g. homework? Average number of hours: ______

DWECS, 2005

(The next questions are about your current work as a wage earner. If you have multiple jobs, we would like you to describe your primary job as a wage earner (the one you use most hours on). If you are not working at the moment, describe your most recent job.)

How many hours a week do you normally work in your primary job, including any established working hours, paid overtime, and other extra work such as work from home?

On average: ______ hours ______ minutes

How many hours per week do you normally work in your second job including any established working hours, paid overtime, and other extra work such as work from home?

On average: ______ hours

DWECS, 2010

How many hours per week do you work in your primary job, including overtime?

Number of hours _______

How many hours per week do you normally work in your second job?

Number of hours _______

COPSOQ, 2004

How many hours per week do you actually work including overtime hours and other jobs?

(Please write the average per week during the last year). ____ hours per week

DANES, 2008

How many hours do you actually work per week in your primary job, including overtime?

(Please write the average per week within the last year) ____ hours per week

If you have a second job, then how many hours per week do you work in your second job? (Please write the average per week within the last year) ____ hours per week

Work schedules:

DWECS, 1995

How are your working hours normally placed?

(Your primary job as a wage earner)

Fixed day duty

Working on two shifts

Working on three shifts

Irregularly placed during the day/week according to special schedule or rotation

Fixed evening shift/evening work

Fixed night shift/night work

Fixed morning duty

Other, please write here

DWECS, 2000

How are your working hours normally placed?

(Your primary job as a wage earner)

Fixed day duty

Working on two shifts

Working on three shifts

Irregularly placed during the day/week according to special schedule or rotation

Fixed evening shift/evening work

Fixed night shift/night work

Fixed morning duty

Other, please write here

DWECS, 2005

How are your working hours normally placed?

(Your primary job)

Fixed day duty

Working on two shifts

Working on three shifts

Irregularly placed during the day/week according to special schedule or rotation

Fixed evening shift/evening work

Fixed night shift/night work

Fixed morning duty

Other

If other, indicate what:

DWECS, 2010

At what time of the day do you usually work in your primary job?

Fixed day duty

Fixed evening work (primarily between 3 pm and midnight)

Fixed night work (primarily between midnight and 5 am)

Variable working hours with night work

Variable working hours without night work

Other, write:

COPSOQ, 2004

At what time of the day do you usually work?

Fixed day duty (primarily between 6 am and 6 pm)

Fixed evening work (primarily between 3 pm and midnight)

Fixed night work (primarily between 10 pm and 6 am)

Variable working hours without night work

Variable working hours with night work

Other, write:

DANES, 2008

At what time of the day do you usually work in your primary job?

Fixed day duty (primarily between 6 am and 6 pm)

Fixed evening work (primarily between 3 pm and midnight)

Fixed night work (primarily between 10 pm and 6 am)

Variable working hours

Other, write:
